# Post-stroke balance rehabilitation under multi-level electrotherapy: a conceptual review

**DOI:** 10.3389/fnins.2014.00403

**Published:** 2014-12-15

**Authors:** Anirban Dutta, Uttama Lahiri, Abhijit Das, Michael A. Nitsche, David Guiraud

**Affiliations:** ^1^DEMAR (INRIA Sophia Antipolis), INRIA, CNRS: UMR5506, Université Montpellier II - Sciences et Techniques, Université Montpellier IMontpellier, France; ^2^Laboratoire d'Informatique de Robotique et de Microélectronique de Montpellier, CNRS: UMR5506, Université Montpellier II - Sciences et TechniquesMontpellier, France; ^3^Electrical Engineering, Indian Institute of TechnologyGandhinagar, India; ^4^Department of Neurorehabilitation, Institute of NeurosciencesKolkata, India; ^5^Department of Clinical Neurophysiology, Göttingen University Medical SchoolGöttingen, Germany

**Keywords:** virtual reality, eye tracking, neuromuscular electrical stimulation, stroke, neurorehabilitation, non-invasive brain stimulation

## Abstract

Stroke is caused when an artery carrying blood from heart to an area in the brain bursts or a clot obstructs the blood flow thereby preventing delivery of oxygen and nutrients. About half of the stroke survivors are left with some degree of disability. Innovative methodologies for restorative neurorehabilitation are urgently required to reduce long-term disability. The ability of the nervous system to respond to intrinsic or extrinsic stimuli by reorganizing its structure, function, and connections is called neuroplasticity. Neuroplasticity is involved in post-stroke functional disturbances, but also in rehabilitation. It has been shown that active cortical participation in a closed-loop brain machine interface (BMI) can induce neuroplasticity in cortical networks where the brain acts as a controller, e.g., during a visuomotor task. Here, the motor task can be assisted with neuromuscular electrical stimulation (NMES) where the BMI will act as a real-time decoder. However, the cortical control and induction of neuroplasticity in a closed-loop BMI is also dependent on the state of brain, e.g., visuospatial attention during visuomotor task performance. In fact, spatial neglect is a hidden disability that is a common complication of stroke and is associated with prolonged hospital stays, accidents, falls, safety problems, and chronic functional disability. This hypothesis and theory article presents a multi-level electrotherapy paradigm toward motor rehabilitation in virtual reality that postulates that while the brain acts as a controller in a closed-loop BMI to drive NMES, the state of brain can be can be altered toward improvement of visuomotor task performance with non-invasive brain stimulation (NIBS). This leads to a multi-level electrotherapy paradigm where a virtual reality-based adaptive response technology is proposed for post-stroke balance rehabilitation. In this article, we present a conceptual review of the related experimental findings.

## Introduction

Stroke, defined as an episode of neurological dysfunction caused by focal cerebral, spinal, or retinal infarction, is a global health problem and fourth leading cause of disability worldwide (Strong et al., 2007; Sacco et al., 2013). One of the most common medical complications after stroke are falls, with a reported incidence of up to 73% in the first year post-stroke (Verheyden et al., 2013). Preliminary results from Marigold et al. (2005) suggest that agility training programs challenging dynamic balance may be more effective than static stretching/weight-shifting exercise programs in preventing falls in the chronic stroke population. Stroke-related ankle impairments, which enhance the probability of falls, include weakness of the ankle dorsiflexor muscles and increased spasticity of the ankle plantarflexor muscles. This leads to the foot drop syndrome that is clinically described as poor ankle dorsiflexion during the swing phase along with a forefoot or flat-foot initial contact in the stance phase. Here, the impact of standing balance on activities of daily living is critical, since balance is associated with ambulatory ability (Patterson et al., 2007) and recovery of gross motor function (Tyson et al., 2007). Toward improving muscle strength and reducing muscle spasticity, we leverage recent advances in rehabilitation technology, particularly Neuromuscular Electrical Stimulation (NMES), for post-stroke standing balance rehabilitation. NMES involves coordinated electrical stimulation of nerves and muscles by continuous short pulses of electrical current and has been shown to improve gait speed in subjects poststroke (Robbins et al., 2006). This hypothesis and theory article first proposes a volitionally controlled NMES system for ankle muscles, which acts as a muscle amplifier to improve adequate ankle movement for upright stance during postural perturbations (Hwang et al., 2009). The proposed NMES approach is based on recent state-of-the-art work in humans that postulated that the neural control of muscles may be modular, organized in functional groups often referred to as muscle synergies (Piazza et al., 2012; Chvatal and Ting, 2013).

**Figure 1:**
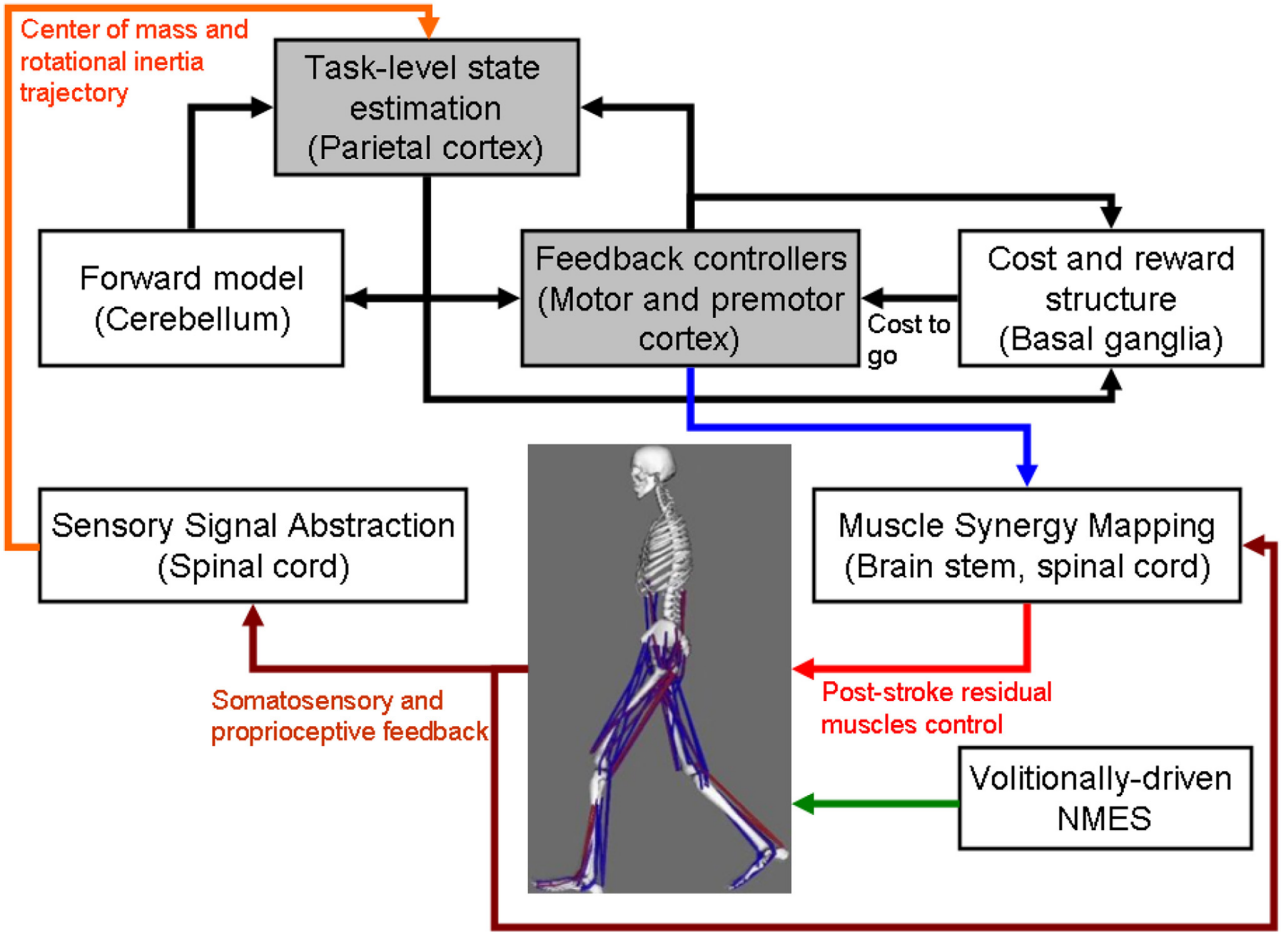
**Computational neuroanatomy for motor control**. Grayed boxes represent the brain regions proposed in this article to be targeted with non-invasive brain stimulation. NMES, neuromuscular electrical stimulation.

During postural perturbations, the body acts as a single segment pendulum centered about the ankle joint where the ankle muscles provide the torque needed to retain upright posture (Hwang et al., 2009). Gatev et al. (1999) presented a feedforward ankle strategy based on the fact that a moderate negative zero-phased correlation exists between the antero-posterior motion of CoP and ankle angular motion. The antero-posterior (A-P) displacements in CoM are performed by ankle plantarflexors (such as medial gastrocnemius and soleus muscles) and dorsiflexors (such as the anterior tibial muscle), while medio-lateral (M-L) displacements are performed by ankle invertors (such as the anterior tibial muscle) and evertors (such as the peroneus longus and brevis muscles) (Winter et al., 1996). Therefore, stroke-related ankle impairments, including weakness of the ankle dorsiflexor muscles and increased spasticity of the ankle plantarflexor muscles, lead to impaired postural control. Respective changes in reflex excitability with respect to postural sway have been shown during standing (Tokuno et al., 2009). For post-stroke standing balance rehabilitation, we thus might be able to ameliorate these stroke-related ankle impairments via an improved modulation of ankle stiffness by modulating muscle tone (Winter et al., 2001) via NMES. We further hypothesize that a coordinated increase in corticospinal excitability of the representation of specific ankle muscles can result in an improved modulation of ankle stiffness. In this connection, prior work has shown that NMES elicits lasting changes in corticospinal excitability, possibly as a result of co-activating motor and sensory fibers (Knash et al., 2003). Moreover, Khaslavskaia and Sinkjaer (2005) showed in humans that concurrent motor cortical drive present at the time of NMES goes along with enhanced motor cortical excitability. Furthermore, at the spinal level, volitionally-driven NMES under visual feedback may induce short-term neuroplasticity in spinal reflexes (e.g., reciprocal Ia inhibition; Perez et al., 2003). Also, corticospinal neurons that project via descending pathways to a given motoneuron pool can inhibit the antagonistic motoneuron pool via Ia-inhibitory interneurons in humans (Pierrot-Deseilligny and Burke, 2005). Consequently, post-stroke impaired reciprocal inhibition between antagonistic muscles may be strengthened via increased presynaptic inhibition of group Ia-afferents under operant conditioning with visual feedback. In this operant conditioning paradigm with visual feedback (Dutta et al., 2013a), the brain acts as the controller during the visuomotor task, where the center of pressure (CoP) is volitionally moved across a display monitor and this movement is assisted with volitionally-driven NMES, as illustrated in Figure 1.

However, prior work suggests that active supraspinal control mechanisms are relevant for balance and their adaptation is important in balance training (Taube et al., 2008). Indeed, supraspinal control mechanisms help to counteract internal perturbations caused by self-initiated movements during activities of daily living to maintain standing balance (Geurts et al., 2005). Balance measures reveal underlying limb-specific control such as between-limb CoP synchronization for standing balance that appears to be a unique index of balance control, independent from postural sway, and load symmetry during stance (Mansfield et al., 2012). A review of standing balance recovery from stroke by Geurts et al. (2005) shows that brain lesions involving particularly the parieto-temporal junction are associated with poor postural control, and suggests that normal multisensory integration in addition to muscle strength is critical for balance recovery. Tokuno et al. (2009) concluded that the sensory feedback mechanisms do relevantly contribute, as the excitability of the respective cortical area was modulated as a function of postural sway, where stroke-related sensorimotor impairment potentially contributes to impaired balance (Mansfield et al., 2012). Indeed, active cortical control based on sensory feedback is relevant for maintaining balance during activities of daily living (Qu and Nussbaum, 2009). In this connection, unilateral spatial neglect, i.e., failure or slowness to respond, orient, or initiate action toward contralesional stimuli, is a common neurological syndrome following predominantly right hemisphere injuries to ventral fronto-parietal cortex (Corbetta and Shulman, 2011). Spatial neglect is associated with prolonged hospital stays, accidents, falls, safety problems, and chronic functional disability (Goedert et al., 2012), probably caused to a relevant degree by compromised cortical control of balance. Here, amelioration of spatial neglect may be possible with non-invasive brain stimulation (NIBS) (Hesse et al., 2011). NIBS—namely transcranial direct current stimulation (tDCS)—over the posterior parietal cortex (PPC) has been shown to modulate visuospatial localization (Wright and Krekelberg, 2014) and to alter perceived position (Wright and Krekelberg, 2013). Moreover, modulation of sensorimotor cortical excitability by tDCS is feasible (Nitsche and Paulus, 2000), and may facilitate post-stroke rehabilitation (Hallett, 2005; Flöel, 2014) by enhancing sensory feedback mechanisms for brain machine interface (BMI) control (Dutta et al., 2014b). Matsunaga et al. (2004) have shown that anodal tDCS over the sensorimotor cortex induces a long-lasting increase of the size of ipsilateral cortical components of somatosensory evoked potentials. Moreover, anodal tDCS enhances corticospinal excitability (Nitsche and Paulus, 2000), including long-term changes of synaptic strength (Nitsche et al., 2008), and anodal tDCS over the primary motor cortex has an impact on spinal network excitability in humans (Roche et al., 2009). Roche and colleagues describe an increase of disynaptic inhibition at the spinal level reflex pathways during anodal tDCS that was caused by an increase in disynaptic interneuron excitability (Roche et al., 2009).

The computational neuroanatomy for motor control (Shadmehr and Krakauer, 2008) is shown in Figure 1. Shadmehr and Krakauer (2008) suggested specific functions of different parts of the brain in motor control. The main function of the

- cerebellum is system identification, i.e., to build internal models that predict sensory outcome of motor commands and correct motor commands through internal feedback.
- parietal cortex is state estimation, i.e., to integrate the predicted proprioceptive and visual outcomes with sensory feedback to form a belief about how the commands affect the states of the body and the environment.
- basal ganglia is related to optimal control, i.e., learning costs and rewards associated with sensory states and estimating the “cost-to-go” during execution of a motor task.
- primary and the premotor cortices are related to implementing the optimal control policy by transforming beliefs about proprioceptive and visual states, respectively, into motor commands.

**Figure 2:**
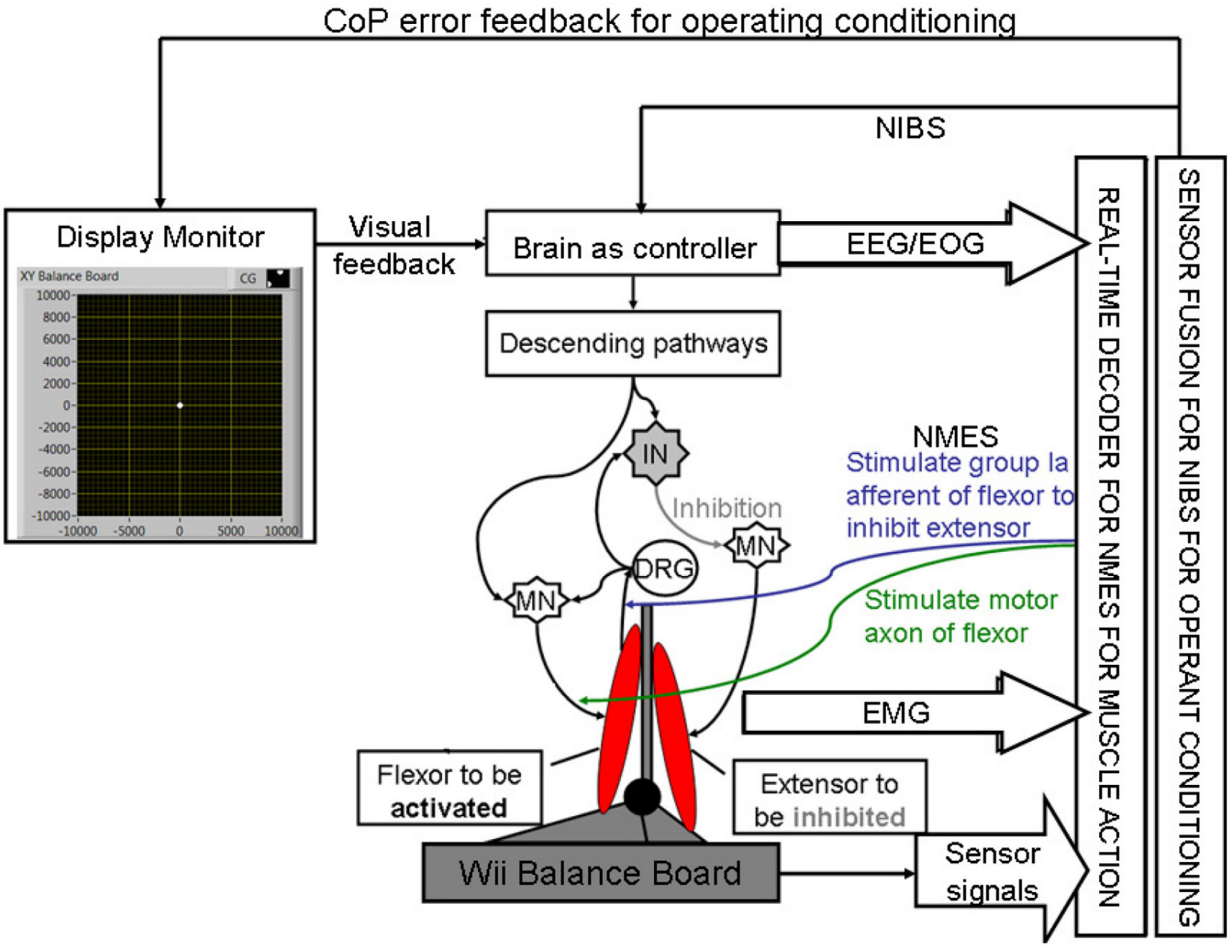
**Schematic drawing of the multi-level electrotherapy paradigm toward operant conditioning to improve ankle flexor-extensor coordination during balance therapy**. Volitionally controlled NMES assists the muscles whereas NIBS assists the brain in cortical control. EEG, electroencephalogram; EOG, electrooculogram; EMG, electromyogram; MN, α-motoneuron; IN, Ia-inhibitory interneuron; DRG, dorsal root ganglion; NMES, neuromuscular electrical stimulation; NIBS, non-invasive brain stimulation; CoP, center of pressure.

Here, during operant conditioning with visual feedback (Dutta et al., 2013a), the brain acts as the controller for trial-by-trial error correction during the visuomotor task which is assisted with volitionally-driven NMES (Figure 1). The real-time decoder for NMES (see Figure 2) acts as a intent detector to assist residual muscle function with electrical stimulation-evoked muscle action. However, stroke survivors often suffer from heterogeneous deficits in cortical control, e.g., delay in initiation and termination of muscle contraction (Chae et al., 2002) as well as deficits in the visuomotor attention networks (Corbetta and Shulman, 2011) conducive for motor learning. Therefore, our hypothesis is that the cortical control of NMES during visuomotor task and motor learning during balance rehabilitation may be facilitated with NIBS. The underlying concept of NIBS approaches is that NIBS can modulate excitability of a targeted cortical region. The sensor fusion for NIBS (see Figure 2) includes a NIBS controller that tries to maintain a more balanced brain state (Schlaug and Renga, 2008). The sensor fusion also includes gaze-interaction with CoP visual feedback (Sailer et al., 2005) to objectively quantify the engagement and stage of motor learning for the affected and unaffected sides, such that the quality of error feedback can be titrated to balance bilateral performance during operant conditioning. The human-machine interface (HMI) integrating biosignal sensors and motion capture with a NMES system for post-stroke balance rehabilitation is based on a point-of-care testing system (Dutta et al., 2013b) that has been shown feasible for EMG-triggered NMES therapy (Banerjee et al., 2014).

## Hypothesis 1: brain acts as a controller for trial-by-trial error correction during visuomotor balance therapy

**Figure 3:**
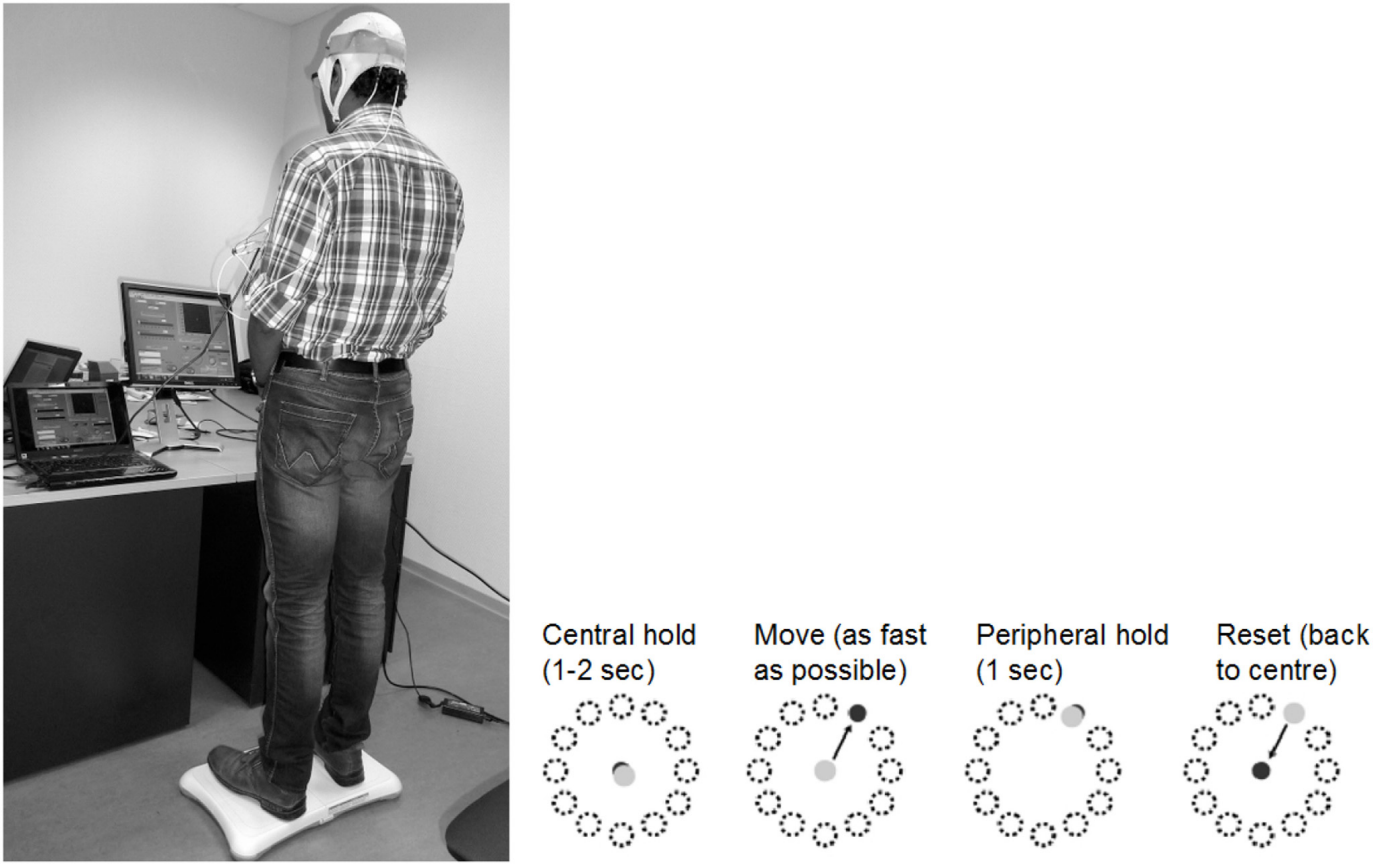
**An example of the visuomotor task where the left panel shows the experimental setup and the right panel shows the modified functional reach task (mFRT)**. The subject is required to steer a computer cursor to a peripheral target using volitionally generated Center of Pressure (CoP) excursions.

As shown in Figure 1, coordinated movement depends on interactions between multiple brain areas leading to transient functional connectivity networks (Shafi et al., 2012) where the brain acts as a controller viz. state estimation, optimization, prediction, cost, and reward. Active participation of motor-cortex (and other cortical areas) may be facilitated by modulating NMES with volitional effort where state-of-the-art prior works show that stimulation envelopes may be controlled (Yeom and Chang, 2010) or triggered (Banerjee et al., 2014) with volitional electromyogram (EMG). During operant conditioning, post-stroke subject volitionally drives NMES during visuomotor task performance for balance rehabilitation where the goal is to reduce error while steering a computer cursor to a peripheral target using volitionally generated CoP excursions, as illustrated in Figure 3. The human machine interface (HMI) integrating biosignal sensors and motion capture for volitionally driven NMES toward operant conditioning with visual feedback was evaluated in a community setting (Banerjee et al., 2014). We present a real-time decoder in Subsection Proposed Method: Volitionally-Driven NMES-Assisted Visuomotor Balance Therapy for volitionally driven NMES that combines physical sensor signals with biopotentials from the HMI to facilitate erect posture recovery following internal postural perturbations caused by self-initiated movements.

A proof-of-concept study (without NMES) on HMI was successfully conducted on 10 able-bodied subjects (5 right-leg dominant males and 5 right-leg dominant females aged between 22 and 46 years) (unpublished material). All subjects gave their written informed consent for the experiments in compliance with the Declaration of Helsinki. They had no known neurological disorder at the time of the study. Here, stroke presents with heterogeneous deficits in motor control where the recovery of erect posture in stroke survivors following CoP excursions is proposed (Subsection Proposed Method: Volitionally-Driven NMES-Assisted Visuomotor Balance Therapy) to be assisted with NMES. Geurts et al. (2005) reviewed cross-sectional studies of voluntary weight-shifting capacity in patients after stroke compared to age-matched healthy control subjects and provided evidence of the following deficits: (1) multi-directionally impaired maximal weight shifting during bipedal standing, in particular toward the paretic leg; (2) slow speed, directional imprecision and small amplitudes of single and cyclic sub-maximal frontal plane weight shifts, most prominently toward the paretic side. An increased magnitude of postural sway has been described for individuals after stroke (Mansfield et al., 2012). Post-stroke sensory deficits may be responsible for these symptoms, because upright standing requires to be stabilized by active control strategies against instability induced by a large neural feedback transmission delay. Mansfield and colleagues proposed that measures of between-limb synchronization, overall postural sway, and weight-bearing symmetry are each independently important measures of post-stroke standing balance control and can reveal discernable control problems (Mansfield et al., 2012).

Prior work suggests that visual CoP feedback during a weight-shifting task may improve performance (Ustinova et al., 2001). Moreover, patients in the post-acute phase of stroke tend to rely more on visual information for postural control in both antero-posterior (A-P) and medio-lateral (M-L) planes than healthy age-matched controls (Geurts et al., 2005). Indeed, excessive reliance on vision for erect standing may decrease during rehabilitation, but can still be found in the chronic phase under more challenging conditions. Such abnormal reliance on vision may be related to a higher-level inability to select the pertinent sensory input. There is evidence that even in the chronic phase of stroke, visual deprivation training can reduce the degree of visual dependence for postural control (Geurts et al., 2005). In accordance, we present an operant conditioning paradigm where CoP excursions steers the cursor on a screen and the visual feedback of the cursor is corrupted by noise thereby effecting visual deprivation. We propose to vary the quality of visual feedback using different noise levels for different locations on the screen according to the visuospatial attention during the visuomotor task for uniform learning of the affected and unaffected sides, and therefore present the subject with a virtual reality toward constrained induced movement therapy (Morris et al., 1997), as discussed in Section Proposed Method: Operant Conditioning Based on Gaze-Interaction in Virtual Reality. In Section Preliminary Evidence: Trial-by-Trial Error Correction during Operant Conditioning, we present evidence from our proof-of-concept study on healthy for trial-by-trial error correction during visuomotor balance therapy under an operant conditioning paradigm.

### Proposed method: volitionally-driven NMES-assisted visuomotor balance therapy

The capacity to voluntarily transfer body weight while maintaining standing balance over a fixed base of support is a prerequisite for safe mobility (Geurts et al., 2005). During balance training, the stroke survivors will voluntary shift their CoP location to steer the cursor as fast as possible under visual feedback. The state-of-the-art prior works show that NMES stimulation envelopes may be controlled (Yeom and Chang, 2010; Zhang et al., 2013) or triggered (Dutta, 2009) with volitional electromyogram (EMG) or electroencephalogram (EEG) (Niazi et al., 2012; Takahashi et al., 2012). However, post-stroke biopotentials often suffer from deficits, e.g., EMG suffers from delays in initiation/termination (Chae et al., 2002) as well as fatigue, and therefore solely biopotentials based control of a NMES-assisted dynamic balance task is challenging where such activation delays may result in falls. Such faults may be alleviated through sensor fusion with physical sensor signals (Dutta et al., 2011). Here, able-bodied muscle activation profiles from EMG can be used to define the NMES templates (Kobetic and Marsolais, 1994) for erect posture recovery where (optimal) muscle synergies (Chvatal and Ting, 2013) can be extracted from the EMG recorded bilaterally from healthy ankle muscles approximately coincident with those targeted for NMES (Piazza et al., 2012; Li et al., 2014) in post-stroke subjects right after presentation of the visual cue. The muscle synergy specifies the coordinated activation of several muscles, and each muscle synergy is expected to get activated during specific perturbation directions (A-P or M-L) and time bins following the visual cue (Torres-Oviedo and Ting, 2007). Recent work in humans demonstrates that the neural control of muscles may be modular, organized in functional groups often referred to as muscle synergies (Chvatal and Ting, 2013). Moreover, Torres-Oviedo and Ting (2007) showed that muscle synergies, i.e., a pattern of task-specific co-activation of muscles, represent a general neural strategy underlying muscle coordination in postural tasks. In fact, the composition and temporal activation of several muscle synergies identified across subjects are consistent with “ankle” and “hip” strategies in human postural responses (Torres-Oviedo and Ting, 2007). Although several studies show how the motor system elegantly circumvents the need to control its large number of degrees of freedom through a flexible combination of motor synergies (Chvatal and Ting, 2013), such a framework has not yet been leveraged for the generation of NMES stimulation patterns. Here, Alessandro et al. (2012) discussed the synthesis and adaptation of effective motor synergies for the solution of reaching tasks which can be leveraged with a reduced-order biped model for NMES template generation (Piazza et al., 2012; Li et al., 2014). To model the performance of a dynamic balance task such as volitional CoP excursions while maintaining standing balance over a fixed Base of Support (BoS), we will apply the “extrapolated center of mass” (xCoM) concept to define the Margin of Stability (MoS) (Hof, 2008). Here, bipedal standing is approximated as an inverted pendulum centered about the ankle joint. Its eigenfrequency (ω_0_) can be computed from the leg length (*l*), i.e., the height of the upper margin of the greater trochanter above the floor,

(1) $$\omega_{0}=\sqrt{\frac{g}{l}}$$

where *g* is the acceleration due to gravity. Therefore, the xCoM location, ${\left[\begin{array}{c} x\\ y \end{array}\right]}_{xCoM}$, can be defined from the CoM projection on the ground, ${\left[\begin{array}{c} x\\ y \end{array}\right]}_{CoM}$,

(2) $${\left[\begin{array}{c} x\\ y \end{array}\right]}_{xCoM}={\left[\begin{array}{c} x\\ y \end{array}\right]}_{CoM}+{\left[\begin{array}{c} \dot{x}/\omega_{0}\\ \dot{y}/\omega_{0} \end{array}\right]}_{CoM}$$

During performance of the bipedal reaching task, the maximum excursion of the xCoM location which does not result in a stepping response can be monitored. This will provide an estimate of the MoS within the BoS for standing balance control. Animal studies have shown that perturbations evoke coordinated long-latency responses that help to return the body to its postural equilibrium (Macpherson and Fung, 1999; Deliagina et al., 2008). A real-time decoder can detect this long-latency responses to control and/or trigger NMES to assist the post-stroke subjects to recover to the erect posture following internal perturbations. NMES is based on the observation of intermittent, ballistic-type corrective movements in healthy humans (Loram et al., 2005) where NMES of the ankle muscles will provide the assistive torque not only to generate basic support (i.e., adequate ankle stiffness) (Hwang et al., 2009) for upright standing but also to provide frequent, ballistic bias impulses for regaining balance from micro falls (Loram et al., 2005).

**Figure 4:**
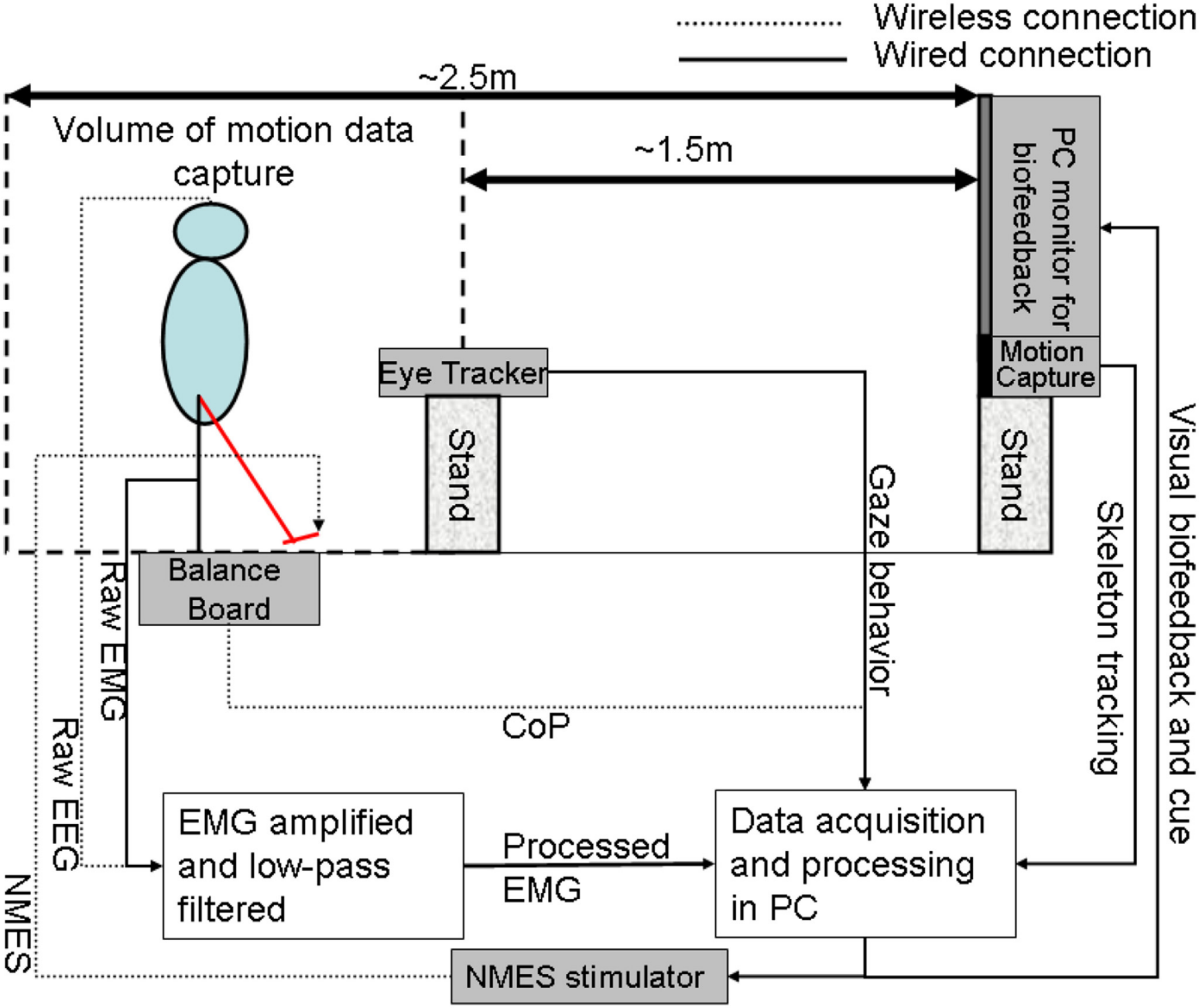
**The human-machine-interface integrating biosignal sensors, eye tracker, and motion capture with a neuromuscular electrical stimulation system for post-stroke balance rehabilitation**. NMES, Neuromuscular Electrical Stimulation; EMG, Electromyogram; EEG, Electroencephalogram; CoP, Center of Pressure; PC, Personal Computer.

In our proof-of-concept study on healthy (no NMES), the augmented HMI system (Figure 4) was used to record CoP-CoM trajectories while the subjects were asked to keep their body rigid, and to maintain full feet contact with the Wii BB. The subjects were asked to lean as far as possible toward forward, toward backward, toward the right side, and toward the left side using visual feedback of the CoP location to provide calibration values for α and β (in Equation 3) such that the cursor does not go off the screen during performance of the visuomotor task when the subject uses full functional reach to steer the computer cursor. During the Central Hold task (CHT), the subjects were asked to keep the cursor close to its origin with CoP excursions, ${\left[\begin{array}{c} x\\ y \end{array}\right]}_{CoP}$, which accelerated the computer cursor, ${\left[\begin{array}{c} \ddot{x}\\ \ddot{y} \end{array}\right]}_{Cur}$, according to Equation (3) (discretized time, *t*, with time-step, *dt*)

(3) $$\begin{array}{l} {\left[\begin{array}{c} \ddot{x}\\ \ddot{y} \end{array}\right]}_{Cur}^{t}=\varepsilon {\left[\begin{array}{c} x\\ y \end{array}\right]}_{CoP}^{t}-\alpha {\left[\begin{array}{c} x\\ y \end{array}\right]}_{Cur}^{t-1}-\beta {\left[\begin{array}{c} \dot{x}\\ \dot{y} \end{array}\right]}_{Cur}^{t-1}+\eta \\ {\left[\begin{array}{c} \dot{x}\\ \dot{y} \end{array}\right]}_{Cur}^{t}={\left[\begin{array}{c} \dot{x}\\ \dot{y} \end{array}\right]}_{Cur}^{t-1}+{\left[\begin{array}{c} \ddot{x}\\ \ddot{y} \end{array}\right]}_{Cur}^{t-1}dt\\ {\left[\begin{array}{c} x\\ y \end{array}\right]}_{Cur}^{t}={\left[\begin{array}{c} x\\ y \end{array}\right]}_{Cur}^{t-1}+{\left[\begin{array}{c} \dot{x}\\ \dot{y} \end{array}\right]}_{Cur}^{t-1}dt \end{array}$$

where ε = 0.01 s^2^ /cm, α = 0.2 s^−2^, β = 0.1 s^−1^, η = *N* (0, σ = 0.1 s^−2^). The visuomotor task was divided into 100 trials lasting for a random duration evenly distributed between 11.5 and 15 s based on prior work of Stevenson et al. (2009). Every 20 ms a new dot was shown on the screen with a position drawn from a radially isotropic Gaussian distribution [*N* (0, 3.5 cm)] centered on the true position of the cursor. The subjects learned to modulate CoP excursion to keep the cursor close to the origin where the mean squared errors (MSEs) were monitored. It was hypothesized that MSE will stay steady during the exploratory stage, show a quick improvement during the skill acquisition stage, followed by a slow improvement during the skill refinement stage. Then, under amodified functional reach task (mFRT) paradigm (Dutta et al., 2014c) in upright standing, called the “Central hold” phase, the subject needs to steer the cursor as fast as possible toward a randomly presented peripheral target as cued by visual feedback (see Figure 3). Following this “Move” phase, the subject will have to hold the cursor at the target location for 1 s during the “Peripheral hold” phase. Following the “Peripheral hold” phase, the cursor will “Reset” back to the center. Following CoP excursion to steer the cursor during the “Move” phase, the recovery of erect posture will be assisted with NMES.

**Figure 5:**
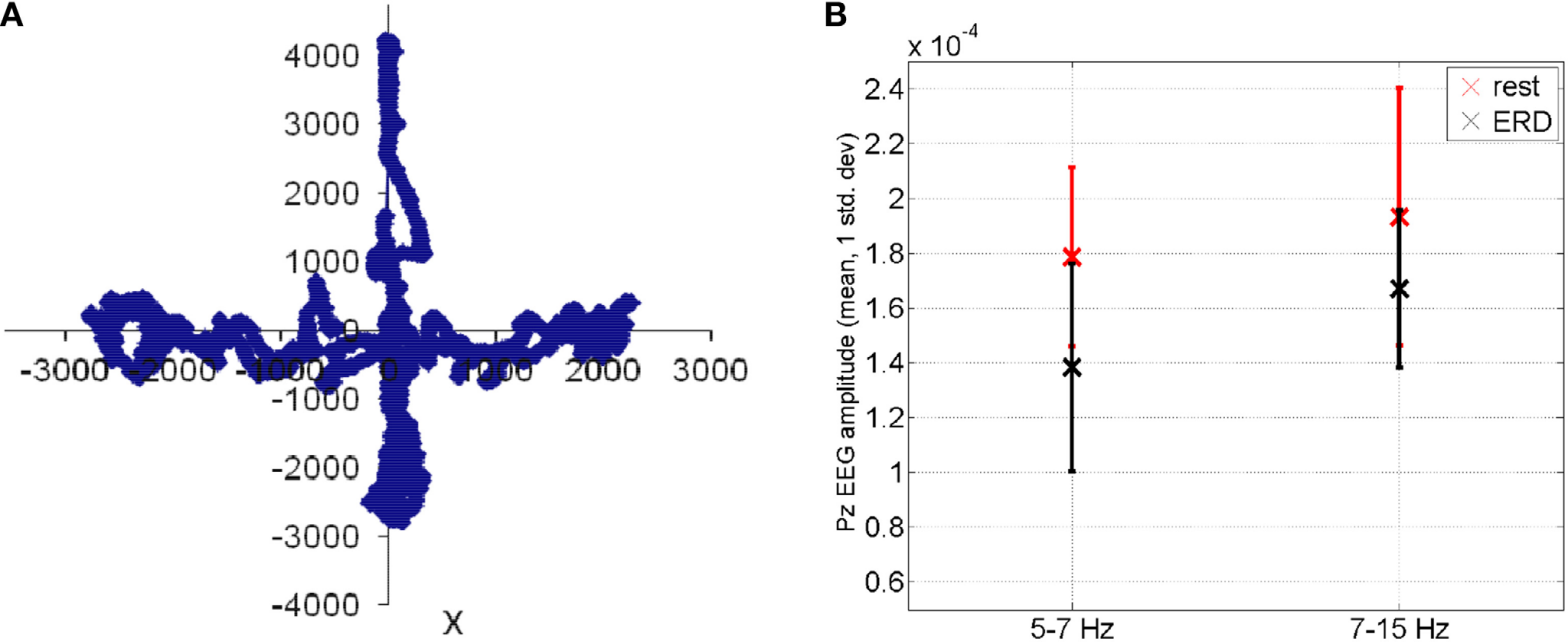
**(A)** During functional reach test, the Center of Pressure was volitionally moved away from the resting state (origin is static equilibrium). **(B)** Feasibility results for event-related desynchronization in able-bodied subjects: EEG amplitude (mean ± std. dev.) at Pz during functional reach test (black color) when compared to the resting state (red color).

In our proof-of-concept study on healthy (no NMES), EEG and electrooculogram (EOG) recordings were investigated to detect motor intent during CoP excursions. EEG recordings were conducted using the Emotiv neuroheadset (Emotiv, Australia) which wirelessly relayed EEG data to the PC from 14 channels (saline soaked sponges of the Emotiv Neuroheadset were replaced with Ag/AgCl electrodes) of the EEG cap (International 10–20 system)—Fp1, Fp2, F3, Fz, F4, C3, Cz, C4, P3, Pz, P4, O1, O2 (plus Common Mode Sense/Driven Right Leg references). EEG electrode impedance was kept below 5 kOhm by scratching the scalp and using conductive gel (Ten20, Weaver, USA). The EEG data were analyzed using EEGlab Matlab (Mathworks, USA) software (Delorme and Makeig, 2004). Additionally, a four-electrode EEG, with one electrode positioned at the outer edge of each eye to monitor the horizontal motions and one electrode positioned above and one below the right eye for obtaining vertical movements, was acquired. The eye-blinks along with saccades were identified using EOGUI Matlab (Mathworks, USA) software [“Eogui—a Software to Analyze Electro-Oculogram (EOG) Recordings - File Exchange - MATLAB Central” 2014^1^], which provides the Duration (milliseconds), Amplitude (angular degree), and Viewing Direction (for the saccades in nautic degree; 0 for upwards gazes, 90 for gaze to the right, 180 for downwards gazes, 270 for gazes to the left). Then, eye-blink artifacts identified from EOG were rejected using EEGlab functions and the artifact-free EEG was analyzed for each trial in 4.096 s epochs using a Hanning time window (epochs were overlapped by 50%), and an estimation of the power spectra was calculated for the absolute alpha (7.5–14 Hz) band via fast Fourier transformation using the Welch technique (“pwelch” in Matlab, MathWorks, USA) to detect alpha event-related desynchronization (aERD) (Pfurtscheller and Lopes da Silva, 1999). aERD appearance was defined when the power was below the resting state value, thereby reflecting cognitive attention during CoP visuomotor task, i.e.,

(4) $$aERD\%=\left(\frac{P_{task}-P_{resting-state}}{P_{resting-state}}\right)\times 100$$

where *P_task_* is the power spectral density estimate during the visuomotor task and *P*_*resting*−*state*_ is the power spectral density estimate during resting state. The mFRT is proposed to quantify the subjects' ability to volitionally shift their CoP position as quickly as possible without losing balance while cued with CoP visual biofeedback. During CHT and mFRT, alpha event-related desynchronization (*aERD*%) was found primarily in the parietal and occipital EEG electrodes (unpublished material), shown by an illustrative plot in Figure 5.

### Proposed method: operant conditioning based on gaze-interaction in virtual reality

The capacity to voluntarily transfer body weight while maintaining standing balance over a fixed base of support is a prerequisite for activities of daily living. Stroke survivors use only a small part of their base of support for voluntary weight displacements. Also, during standing and antero-posterior (A-P) weight-shifting, stroke patients deviate from the mid-line of the base of support more than healthy control subjects (Goldie et al., 1996). Moreover, compared to control subjects, stroke patients have significant deficits in the ability to weight-shift in the medio-lateral (M-L) direction (Goldie et al., 1996). Furthermore, there is strong evidence that physiological markers such as blink rate can be used as effective indicator of one's mental workload (Marshall, 2007). In our augmented HMI, two Wii Balance Board™ (Wii BB) (Nintendo, USA) (Clark et al., 2010) were positioned side by side without touching (i.e., <1 mm apart). Following the experimental protocol of Mansfield and colleagues (Mansfield et al., 2012), the subjects could stand with one foot on each Wii BB in a standard position (feet oriented at 14° with 7° rotation of each foot with an inter-malleoli distance equal to 8% of the height), with each foot equidistant from the midline between both Wii BBs. In our integrated system, we augment the operant conditioning paradigm with a gaze-sensitive virtual reality-based adaptive response technology (Lahiri et al., 2013) that evaluates motor learning during the performance of the visuomotor task with regard to visuomotor coordination via applying the principles of engagement. Specifically, making the visuomotor task easier for the affected side in virtual reality may yield greater neuroplastic changes and functional outcomes in neurorehabilitation (Danzl et al., 2012).

The post-stroke subject will stand with a minimum baseline stimulation level necessary to generate basic support for upright standing according to clinical observation. From this upright standing, the patient needs to steer the cursor as fast as possible toward a randomly presented peripheral target as cued by visual feedback (see Figure 3) under a modified functional reach task (mFRT) paradigm, as discussed in Section Proposed Method: Operant Conditioning Based on Gaze-Interaction in Virtual Reality. During the bipedal reaching task using visual feedback, the acceleration of the cursor can be controlled with CoP excursions measured by two (one for each limb) Wii BB according to the following dynamics (Stevenson et al., 2009),

(5) $$\begin{array}{l} {\left[\begin{array}{c} \ddot{x}\\ \ddot{y} \end{array}\right]}_{Cur}=\varepsilon_{1}{\left[\begin{array}{c} x\\ y \end{array}\right]}_{CoP_{1}}+\varepsilon_{2}{\left[\begin{array}{c} x\\ y \end{array}\right]}_{CoP_{2}}\\ \;-\alpha {\left[\begin{array}{c} x\\ y \end{array}\right]}_{Cur}-\beta {\left[\begin{array}{c} \dot{x}\\ \dot{y} \end{array}\right]}_{Cur}+\eta \end{array}$$

where ε_1_, ε_2_ parameterizes the effect of recorded *CoP*_1_, *CoP*_2_ excursions ${\left[\begin{array}{c} x\\ y \end{array}\right]}_{CoP_{1}}$ and ${\left[\begin{array}{c} x\\ y \end{array}\right]}_{CoP_{2}}$ on the cursor acceleration, ${\left[\begin{array}{c} \ddot{x}\\ \ddot{y} \end{array}\right]}_{Cur}$, and α and β parameters prevent the cursor from going off-screen, and η ~ *N* (0, σ_*p*_) represents the process noise with variance σ_*p*_. The increase in gain ε_1_, ε_2_ makes the task require lesser CoP excursion range while a decrease in the variance, σ_*p*_, reduces the uncertainty. Task difficulty can be increased by decreasing the gain ε_1_, ε_2_ and increasing the variance, σ_*p*_, where we present the subject with a virtual reality toward constrained induced movement therapy (Morris et al., 1997). Furthermore, toward constrained induced movement therapy (Morris et al., 1997), visual deprivation will be effected by introducing observer noise in the visual feedback by flashing a low contrast dot on the screen with a position drawn from a radially isotropic Gaussian distribution centered on the true position of the cursor. The variance representing this Gaussian cloud of points *N* (0, σ_*o*_), will introduce observer noise as shown by prior work (Stevenson et al., 2009). Therefore, task difficulty can be modulated with parameters ε_1_, ε_2_, σ_*p*_, and σ_*o*_ for the affected and unaffected limbs during operant conditioning. For example, the gain, ε_2_, ε_2_, can be set individually for the affected and unaffected limbs for each peripheral target such that they present similar reaching errors during the exploratory stage of motor learning for the unipedal reaching task, which may lead to comparable reward expectations. During performance of the bipedal reaching task, the subject can learn to volitionally modulate CoP excursions using coordinated bipedal muscle activity to generate cued cursor movement under visual feedback. Here, identification of visuospatial attention and motor learning is critical for constrained induced movement therapy (Morris et al., 1997) where a Bayesian framework addresses the problem of updating beliefs and making inferences based on observed data. We present a standard Kalman filter to compute the estimated state of the cursor from observations while capturing user behavior during the reaching task, i.e., the “Central hold,” “Move,” and then “Peripheral hold” phases of the task. The peripheral targets are at the subject-specific limits (position and velocity) of CoP excursion, which are mapped using the α and β parameters of the Equation (5) for each target. An important feature of the Kalman filter is how estimation changes as a function of feedback uncertainty (Stevenson et al., 2009). For example, increasing the observation noise by increasing the variance, σ_*o*_, for a certain peripheral target while keeping the process dynamics and process noise identical (ε_1_, ε_2_, σ_*p*_) may have different effects on its state updates (i.e., Kalman update) based on post-stroke residual function. Hence, the Kalman filter model allows to interrogate the post-stroke control mechanisms by capturing the effects of observation noise (or, visual feedback uncertainty) on the control of cursor state (and reaching errors) toward multi-directional peripheral targets. The Kalman filter assumes that the cursor state, *X* = [*x*, *y*, $\dot{x}$, $\dot{y}$], at time *t* evolves from the state at time, *t* − 1, according to linear dynamics and control:

(6) $$X_{t}\;=AX_{t-1}\;+Bu_{t-1}\;+W_{t}$$

where *u_t_* is the control signal and *W_t_* is the process noise derived from a Gaussian distribution. Here, A and B follow from the Equation (6) for an ideal observer and *W_t_* reflects the effects of process noise η ~ *N* (0, σ_*p*_). For example, Stevenson et al. (2009) found bang-bang controller more similar to human control mechanisms than a linear-quadratic regulator (LQR) during bipedal reaching tasks.

(7) $$u_{t}=\lambda_{1}sign\left(\left[\begin{array}{cc} \cos\theta & \sin\theta \end{array}\right]X\right)+\lambda_{0}$$

Here, θ parameterizes the decision rule for a given state of the cursor, *X*, and λ_0_, λ_1_ parameterizes the magnitude of the two states for bang-bang control for each peripheral target, to capture the “Move” phase toward that target. Moreover, Stevenson et al. (2009) have found that healthy humans readily dampen the cursor oscillations induced by the process noise, η ~ *N* (0, σ_*p*_) which may be deficient post-stroke based on residual function. Here, the cross-correlation between the fluctuations of cursor dynamics (process noise, η ~ *N* (0, σ_*p*_) and CoP during the “Central hold” and “Peripheral hold” phases of the task should be consistent with ideal observer models in normal cases of residual function, i.e., subjects should respond more slowly and with lower amplitudes when the feedback is more uncertain (increased variance, σ_*o*_). Also the gains, ε_1_, ε_2_, i.e., the range of CoP excursion required for the reaching task, should not affect subject's control policy in normal cases of residual function (Stevenson et al., 2009). Therefore, the post-stroke subject's postural control policies can be evaluated for each peripheral target by changing the gain to investigate if hemiparesis affects control strategies for reaching certain targets, e.g., at the paretic side. Especially at low gains, the visuomotor task becomes challenging when the subject may use compensatory mechanisms to map between CoP actions and their visual sensory consequences. Motor learning will start from exploratory and skill acquisition to skill refinement stages. The reach errors will stay steady during the exploratory stage and will show a quick improvement during the skill acquisition stage followed by slow improvement during the skill refinement stage. In fact, this can be detected with gaze behavior where active sensing with eye movements during exploratory actions may contribute to coupling of perception and action (Vickers, 2009). For example, the quiet eye (QE) period can be defined as the elapsed time between the last visual fixation to the target and the initiation of the motor response, which has emerged as a characteristic of higher levels of performance (Vickers, 2009). In fact, Mann et al. (2007) presented a meta-analysis that supported the critical role of visual attention in the expert advantage, revealing that experts consistently exhibit fewer fixations of longer duration than non-expert comparison groups. Moreover, during visuomotor tasks, Mann et al. (2011) found that experts exhibit a prolonged QE period and greater cortical activation in the right-central region compared with non-experts.

Prior work on gaze behavior during eye-hand coordination (Sailer et al., 2005) suggests that gaze interaction can provide an evaluative feedback of motor learning. It starts with pursuing the cursor during the exploratory stage, continues with predicatively marking the desired cursor positions during skill acquisition stage, and ends up with direct shifts toward the target during the skill refinement stage. Therefore, the time delay, τ_*delay*_, between the two signals, as determined by the argument of the maximal cross-correlation, should indicate the stage of motor learning. Moreover, during skill acquisition the desired cursor trajectory can be decoded from gaze activity to see if the desired cursor positions are successfully reached under visuomotor control during the “Move” phase. Here, Bayesian learning involves computing the posterior probability distribution of the stage of motor learning during the “Move” phase from the observed gaze-interaction (i.e., τ_*delay*_) where a coarse estimate of the stage of motor learning is based on the reach error at the end of the “Move” phase. In this center-out bipedal visuomotor reaching task with zero process and observer noise (σ_*p*_ = 0 and σ_*o*_ = 0), two modes of performance—skilled, unskilled—are possible during the “Move” phase. These two modes of performance (or hypotheses) are considered to be mutually exclusive and exhaustive hypotheses, H = {*h_skilled_*, *h_unskilled_*}, and can be formulated for each cued peripheral target, *T_i_*, during the visuomotor reaching task. In the Bayesian framework, we denote the degree of belief in a hypothesis by probabilities and determine this belief, called posterior probabilities, using the product of data likelihood and prior probabilities (Bayes's rule):

(8) $$P(h_{i}|d,T_{i})=\frac{P(d|h_{i},T_{i})P(h_{i})}{\sum_{i\;=\;1}^{N}P(d|h_{i},T_{i})P(h_{i})}$$

where prior probabilities, *P*(*h*_*i*_), represent the belief before observing the data, *d* (e.g., τ_*delay*_, etc.), and likelihoods, *P*(*d*|*h_i_*, *T_i_*), for each peripheral target, *T_i_*, denote the probability with which we would expect to observe the data if the hypothesis is true. To estimate the best peripheral targets for motor learning (i.e., distinguishing *h_skilled_*, *h_unskilled_*) with subject-specific gaze interaction data, *d*, the confusion probability matrix for each possible peripheral target, *T_i_*, can be found

(9) $$C_{T_{i}}=\int \sqrt{p(d|h_{skilled},T_{i})p(d|h_{unskilled},T_{i})dd}.$$

Here, we present a modular neural network implementing Bayesian learning and inference for each possible peripheral target, *T_i_*, as described in a prior work by Kharratzadeh and Shultz (2013).

The first module, Module 1, implements the Bayes's rule assuming that the values of prior and likelihood probabilities are given as input. Its output is the posterior probability. This needs to be run for each hypothesis.

Module 2 computes the likelihood probabilities based on observed data. The role of Module 2 is to learn these distributions as the underlying mechanisms generating the data. For example, positive τ_*delay*_ (i.e., gaze pursuing the cursor positions) represents unskilled performance, i.e., at the exploratory stage and negative τ_*delay*_ (i.e., gaze predicatively marking the desired cursor positions) indicates skilled performance, i.e., at the end of skill acquisition stage. Here, the generative process can be described as a Gaussian with mean *h_i_* and standard deviation 1; with positive mean for *i* = *unskilled*, negative mean for *i* = *skilled* (Kharratzadeh and Shultz, 2013),

(10) $$f(d,h_{i})=\frac{1}{2\pi }e^{\frac{{\left(d\;-\;h_{i}\right)}^{2}}{2}}$$

Module 3 computes the hypothesis's prior probabilities by learning their generative discrete distribution function. For example, the generative function can be of the form (Kharratzadeh and Shultz, 2013),

(11) $$g(h_{i})=\upsilon e^{\frac{h_{i}^{2}}{2}}$$

where υ is chosen such that the sum of the prior probabilities equal 1.

For our hypotheses presented for each peripheral reach target, we need to first learn Modules 1 and 3 one-time based on the gaze behavior with respect to the cursor position during the “Move” phase toward a peripheral reach target. During exploratory and skill acquisition stages of operant conditioning, multiple “Move” phases will be performed for each peripheral reach target where the stage of motor learning can be estimated from the reach error following each “Move” phase (Stevenson et al., 2009). A variant of the cascade correlation method, called the sibling descendent cascade correlation (SDCC), can be used to train the Modules using input(s)-output training pairs as shown earlier (Kharratzadeh and Shultz, 2013). After learning Modules 1 and 3, we will use them twice for computing the posterior and prior of each hypothesis, which needs to be learned for each hypothesis, using Module 2. After sufficient training on gaze-interaction, the modular neural network will provide online feedback of the subject's stage of motor learning during the “Move” phase toward the cued peripheral reach target, *P*(*h_i_*|*d, T_i_*), that is based on the observed saccades and gaze fixations with respect to the cursor position (Equation 8). Here, the confusion matrix, *C_T_i__*, will provide an estimate of the subject-specific performance of such a classifier (Equation 9). Therefore, a comparable reach error can be maintained across peripheral reach targets by online adaptation of σ_*p*_ for operant conditioning of volitional multi-directional CoP excursions. For example, increasing the variances, σ_*p*_, will increase the difficulty of the visuomotor task during the “Move” phase, leading to an increase in the reach error at the end of the “Move” phase. Such performance-based adaptive schedules have been shown to enhance motor learning when compared to random scheduling (Choi et al., 2008).

Based on these prior investigations and specifically on a prior work on gaze behavior during eye-hand coordination (Sailer et al., 2005), we postulate that gaze interaction may provide evaluative feedback of motor learning during the “Move” phase that can be used to adapt cursor dynamics such that compensatory mechanisms of the unaffected side can be constrained (Taub and Morris, 2001) toward constrained induced movement therapy (Morris et al., 1997). Such performance-based adaptive schedules have been shown to enhance motor learning when compared to random scheduling (Choi et al., 2008).

### Preliminary evidence: trial-by-trial error correction during operant conditioning

**Figure 6:**
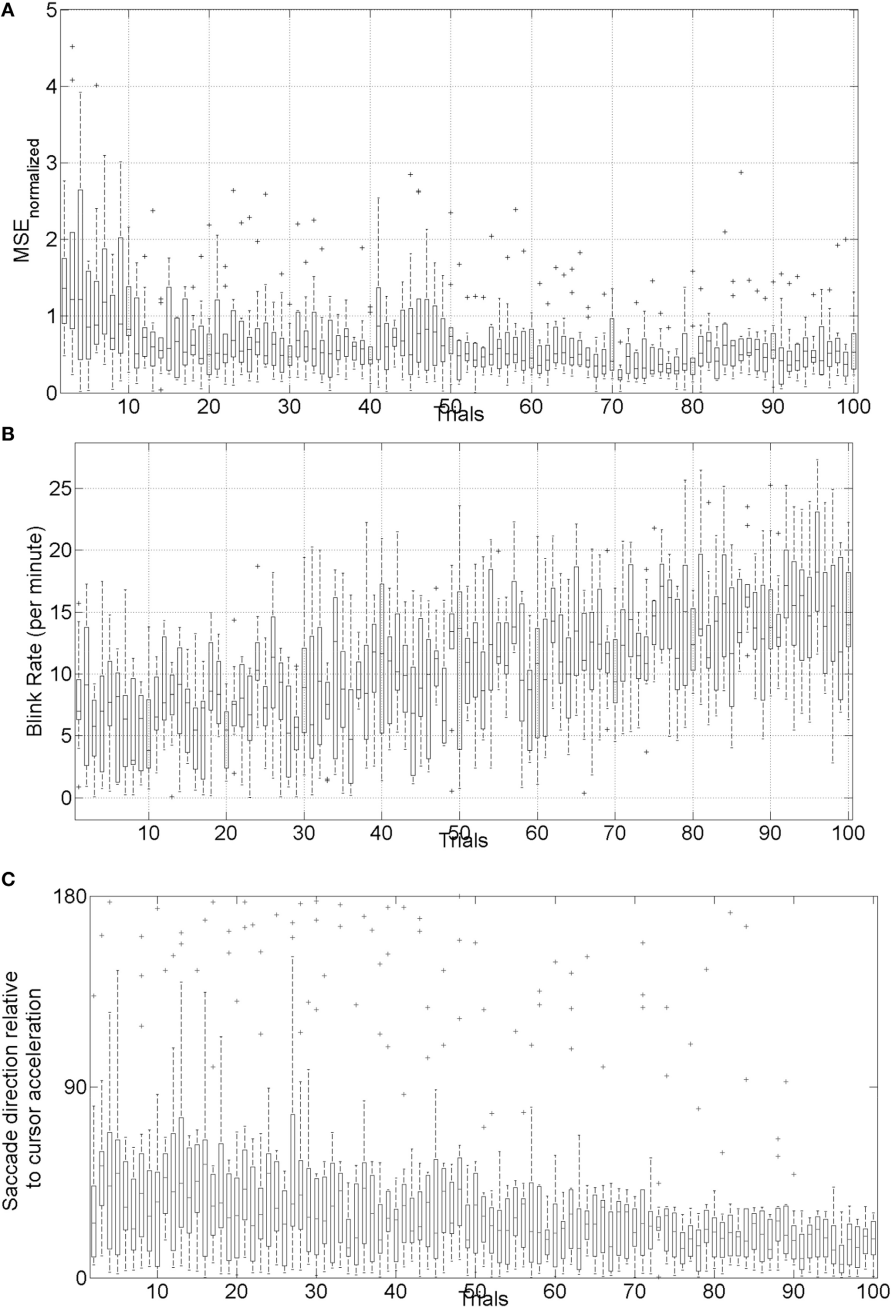
**(A)** Box-plot of normalized mean squared error (MSE) across 10 subjects, **(B)** box-plot of the blink rate (blinks per minute) during the visuomotor task, **(C)** box-plot of saccadic direction relative to the cursor acceleration during the visuomotor task.

**Figure 7:**
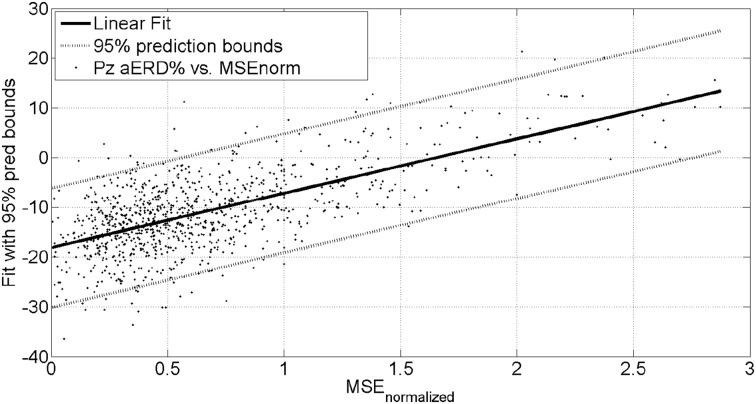
**Alpha event-related desynchronization (aERD%) at Pz EEG electrode vs. baseline normalized mean squared error (MSEnorm) during the visuomotor task performance**. A linear fit with 95% prediction bounds is additionally depicted.

The mean square error (MSE) and gaze-interaction (Sailer et al., 2005) with the visual feedback can be continuously monitored during the visuomotor task and post-stroke subjective learning in the affected and unaffected sides may be modulated by changing the respective error feedback in an operant conditioning framework (Dutta et al., 2013a), i.e., in principle constrained induced movement therapy (Morris et al., 1997) in a virtual reality. A conceptual review of this operant conditioning framework for balance training is presented in the last Subsection Proposed Method: Operant Conditioning Based on Gaze-Interaction in Virtual Reality. Additionally, modulation of event-related desynchronization (ERD) with motor cortex tDCS in healthy volunteers (Matsumoto et al., 2010) and patients with chronic severe hemiparetic stroke has been shown feasible (Kasashima et al., 2012). Based on our proof-of-concept study on healthy (no NMES), the MSE normalized by the baseline value [(without process noise, i.e., η = *N* (0, σ = 0*s*^−2^)] trended toward a decrease (see Figure 6), the blink rate trended toward an increase (see Figure 6), and the saccadic direction relative to the cursor acceleration trended toward zero (see Figure 6) during the visuomotor task under an operant conditioning paradigm. Moreover, Figure 7 shows that the *aERD*% at the position Pz correlated with the normalized mean square error (MSEnorm) during the visuomotor task performance in CHT. The 95% prediction bounds are also shown for a linear-fit which indicates a 95% chance that a new observation is placed within the lower and upper prediction bounds. The coefficients (with 95% confidence bounds) of the linear fit, *aERD*% = *a* × *MSE_normalized_* + *b*, are *a* = 10.97 (10.19, 11.76) and *b* = −18.16 (−18.8, −17.52). The *R*^2^-value was 0.4316 indicating the goodness of fit. Moreover, during mFRT, we could correctly classify roughly 76% of the movement directions as left or right based on pair-wise *aERD*% asymmetry in P3, P4, and O1, O2 electrodes from epochs lasting 0 to 700 ms following peripheral cue presentation (unpublished material).

Therefore, MSE and gaze-interaction (Sailer et al., 2005) can be continuously monitored during the visuomotor task and poststroke subjective learning in the affected and unaffected sides may be modulated by changing the respective error feedback in an operant conditioning framework (Dutta et al., 2013a), i.e., in principle constrained induced movement therapy (Morris et al., 1997) in a virtual reality. Additionally, modulation of event-related desynchronization (ERD) with motor cortex tDCS—a NIBS modality—in healthy volunteers (Matsumoto et al., 2010) and patients with chronic severe hemiparetic stroke has been shown feasible (Kasashima et al., 2012). Also, tDCS over PPC has been shown to modulate visuospatial localization (Wright and Krekelberg, 2014) where lesions in human PPC can lead to complex syndromes consisting of an inability to attend, perceive and react to stimuli in the visual field contralaterally to the lesion, an inability to voluntarily control the gaze, and an inability to coordinate visually elicited hand movements (Caminiti et al., 2010; Lindner et al., 2010; Hwang et al., 2012). Based on these prior works, we postulate that NIBS can facilitate trial-by-trial error correction process during visuomotor balance therapy under operant conditioning.

## Hypothesis 2: NIBS can facilitate trial-by-trial error correction and its retention during operant conditioning

In Subsection Preliminary Evidence: Trial-by-Trial Error Correction during Operant Conditioning, evidence for trial-by-trial error correction during operant conditioning was presented where the brain acting as a controller need to not only perform trial-by-trial error correction but also need to adapt in response to prior error information via retention which is called motor adaptation. Here, active participation of cortical areas may be facilitated with NIBS of motor and premotor cortex, cerebellum, and/or PPC (see Figure 1). A hierarchical error processing was proposed (Krigolson and Holroyd, 2007) in the brain acting as a controller where error processing during visuomotor control involves the evaluation of “high-level” errors (i.e., failures to meet a system goal) by a frontal system involving the anterior cingulate cortex and the basal ganglia (Krigolson and Holroyd, 2006; Holroyd and Coles, 2008), and the evaluation of “low-level” errors (i.e., discrepancies between actual and desired motor commands) by a posterior system involving the PPC and/or the cerebellum (Desmurget and Grafton, 2000; Pisella et al., 2000; Miall et al., 2001; Gréa et al., 2002). Here, the PPC is an important interface between sensory and motor cortices, integrating multimodal sensory and motor signals to process spatial information for a variety of functions including guiding attention and planning movements (Andersen and Gnadt, 1989; Snyder et al., 1997).

In our single-blind, sham-controlled study (Dutta et al., 2014c), five healthy right-leg dominant subjects (age: 26.4 ± 5.3 yrs) were evaluated using the HMI system under two conditions—with anodal tDCS of primary motor representations of right tibialis anterior muscle and with sham tDCS. Paired *t*-test (Matlab “ttest” function, The Mathworks, Inc., USA) was performed for the differences in % change of stabilogram metrics from baseline values after administrating tDCS/sham session, for all the subjects pooled together. The results showed that anodal tDCS of primary motor representations of the right tibialis anterior muscle strongly (*p* < 0.0001) affected maximum CoP excursions but not return reaction time in healthy volunteers. Also, anodal tDCS had a strong (*P* < 0.0001) effect on the % change (decrease) in sway area from baseline values when compared to sham at 45 and 60 min post-tDCS. Anodal tDCS had only a moderate effect (*P* = 0.0113) on the change (decrease) in the path length of the CoP trajectory from the respective baseline value when compared with sham 60 min post-tDCS. Moreover, the results showed that anodal tDCS strongly (*P* < 0.0001) affected the change in centroid of CoP data-points from baseline value during quiet standing in the medio-lateral direction when compared to sham tDCS. The reason for this change in the centroid of CoP data-points during quiet standing (Dutta et al., 2014c) following motor cortex tDCS is postulated to be inadvertent parietal tDCS due to the active electrode position roughly 1 cm left lateral and 2 cm posterior to Cz (International 10-20 EEG system), i.e., close to P3, and relatively high current density (0.06 mA/cm^2^). Indeed, the PPC is an important interface between sensory and motor cortices, integrating multimodal sensory and motor signals to process spatial information for a variety of functions including guiding attention and planning movements (Andersen, 1997). Therefore, a tDCS protocol targeting the PPC is presented in the Subsection Proposed Method: NIBS Protocol to Facilitate Trial-by-Trial Cortical Control and Adaptation During Visuomotor Task. Here, in order to test successful trial-by-trial error correction and its retention during visuomotor balance therapy under operant conditioning, we propose in Subsection Proposed Method: Using Aftereffects to Evaluate Successful Trial-by-Trial Adaptation During Operant Conditioningthe use of aftereffects that occur in motor control when the visual or mechanical variables of the targets are perturbed in a systematic manner. This is based on our prior work on using aftereffects to evaluate successful adaptation during EMG-driven NMES-assisted locomotor exploration activity for post-stroke gait training (Dutta et al., 2014a) where we found that only stroke subjects who showed aftereffects during systematic perturbation of the “EMG to NMES mapping” parameters at random catch-trials during the locomotor exploration activity, showed post-intervention changes in the EMG pattern during volitional (no NMES) treadmill walking.

### Proposed method: NIBS protocol to facilitate trial-by-trial cortical control and adaptation during visuomotor task

Analysis of simultaneously acquired EEG/EMG and gaze-interaction data can be used to assess potential mechanisms underlying skill acquisition during visuomotor task (Mann et al., 2011). During volitionally generated CoP excursions based on visual feedback (Figure 3), the visual system must orient to and process the relevant visual (target) cues to ascertain both distance and direction of the required CoP excursion, while the working memory is called upon for the required joint torques to match the cursor with the visual (target) cues. Recent investigations lend support to the motor programming/preparation function of the QE period based on simultaneous EEG recordings (Mann et al., 2011) where slow cortical potential (SCP) negative shifts in EEG preceding voluntary movement, called bereitschaftspotential (BP) (Shibasaki and Hallett, 2006), lends itself well to the study of the preparatory period preceding task execution. Indeed, Mann et al. (2011) found: (1) greater BP negativity (particularly in central recording locations) for the expert compared with non-experts, and (2) QE duration was associated with BP negativity in central cortical regions. Therefore, it was postulated that the QE is a temporal period when task-relevant environmental cues are processed and motor plans are coordinated for the successful completion of an upcoming task. In our preliminary study (Dutta, 2014), we found that motor cortex anodal tDCS: (1) increased the frequency of negative epochs of the early (2.5 s–300 ms) phase of SCP before movement initiation, and (2) the slope of negative epoch for the late (300 ms–0 s) phase of SCP before movement initiation. Our NIBS protocol to facilitate cortical control and adaptation is based on the hypothesis that throughout the preparation and movement phases of skill execution, the visual attention centers (i.e., occipital and parietal cortex) disseminate requisite commands to motor regions of the cortex (i.e., motor cortex, premotor cortex, supplementary motor area, basal ganglia, and cerebellum), each of which are reflected in BP components (Mann et al., 2011). Here, our preliminary results from healthy subjects on facilitating myoelectric-control with tDCS (Dutta et al., 2014b) showed specific, and at least partially antagonistic effects, of motor cortex and cerebellar anodal tDCS on motor performance during myoelectric control where cerebellum may play a critical role in both formation of motor memory and its retention (Herzfeld et al., 2014). Moreover, during visuomotor task performance, visual search to orient to and process the relevant visual (target) cues require contributions of human frontal eye fields (FEF) and PPC where PPC seems to be involved only when a manual motor response to a stimulus is required (Muggleton et al., 2011). Therefore, PPC may play a critical role in the preparatory activity in the general context of sensorimotor transformations linking perception to action where the SCP (e.g., BP) reflects activation of subcortical and cortical generators (cortico-basal ganglia-thalamo-cortical circuitry) necessary not only in motor execution but also in its preparation (Jahanshahi and Hallett, 2003).

**Figure 8:**
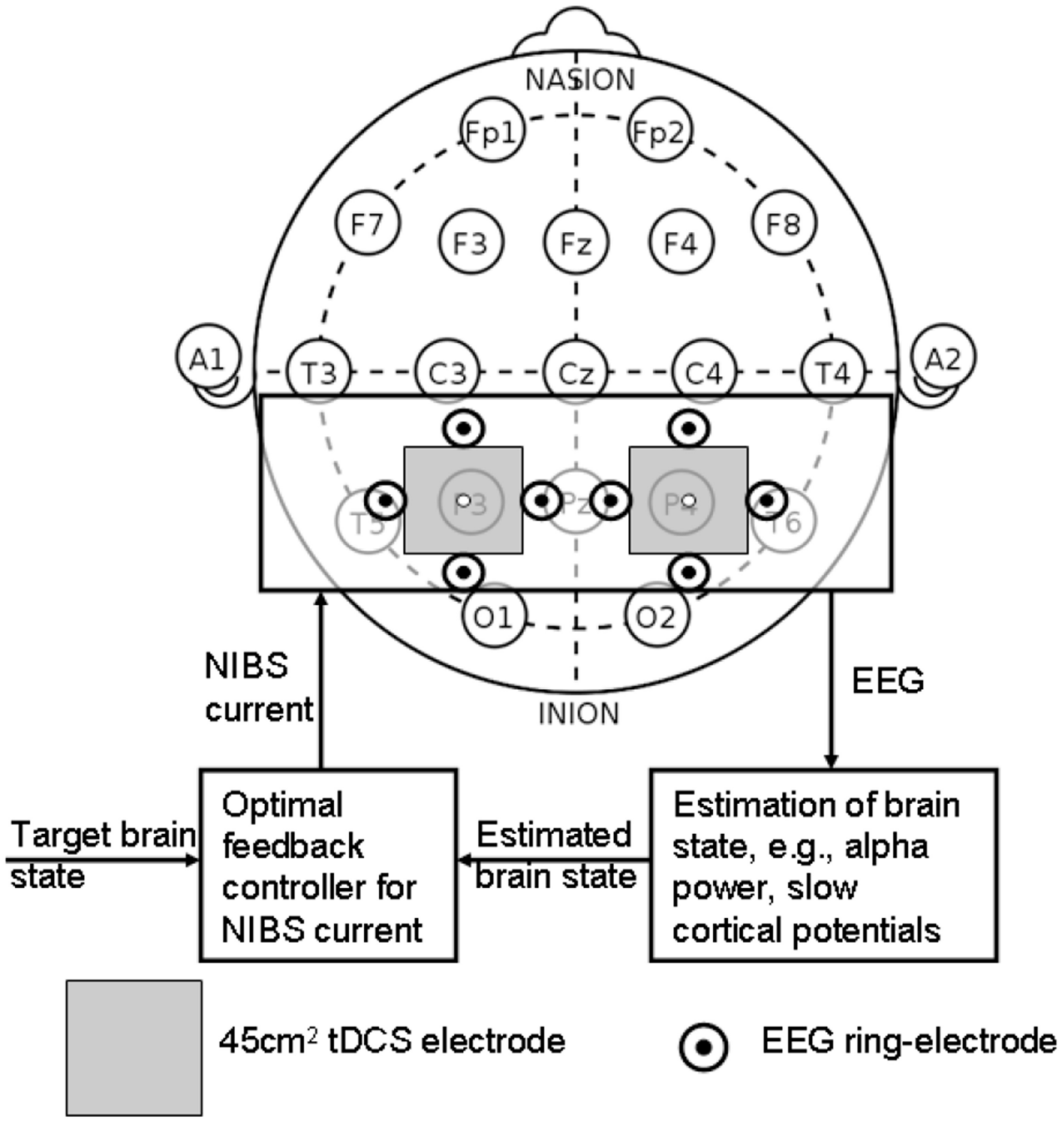
**Closed-loop feedback control of bihemispheric tDCS for posterior parietal cortex**.

Wright and Krekelberg (2014) hypothesized that each hemisphere biases processing to the contralateral hemifield and that the balance of activation between the hemispheres contributes to position perception. They presented a bihemispheric tDCS protocol for PPC and hypothesized that excitability is reduced beneath the cathode and increased beneath the anode where closed-loop feedback control of bihemispheric tDCS for PPC using the MatNIC and StarStim (Neuroelectrics, Spain) NIBS interface is presented in Figure 8. Indeed, when Wright and Krekelberg (2013) applied tDCS bilaterally, e.g., cathodal stimulation over right PPC concurrent with anodal stimulation over left PPC (right-cathodal) or vice versa (left-cathodal), they found that both tDCS conditions altered perceived position to the left relative to a sham stimulation baseline condition. This effect was stronger for right-anodal than for right cathodal tDCS, and lasted for about 15 min after stimulation. Based on these prior works, we postulate that bihemispheric application of tDCS (Wright and Krekelberg, 2013) at P3 and P4 (International 10–20 system) will facilitate cortical control during visuomotor task while cerebellar tDCS (Herzfeld et al., 2014) will facilitate up- or down-regulation of error-dependent motor learning and retention in a polarity-dependent manner. Bihemispheric application of tDCS (Wright and Krekelberg, 2013) at P3 and P4 (International 10-20 system) in conjunction with cerebellar tDCS (Herzfeld et al., 2014) is postulated to facilitate cortical control and adaptation during visuomotor task performance especially on the affected side since the evaluation of “low-level” errors (i.e., discrepancies between actual and desired motor commands) is hypothesized to be performed by a posterior system involving the PPC and/or the cerebellum (Desmurget and Grafton, 2000; Pisella et al., 2000; Miall et al., 2001; Gréa et al., 2002).

### Proposed method: using aftereffects to evaluate successful trial-by-trial adaptation during operant conditioning

Trial-by-trial error correction during the visuomotor task may be facilitated with bihemispheric application of tDCS for PPC (Wright and Krekelberg, 2013). However, it is important to also evaluate trial-by-trial motor adaptation during the visuomotor task under operant conditioning paradigm that may be facilitated with cerebellar tDCS (Herzfeld et al., 2014). Here, Held and colleagues (Held and Gottlieb, 1958; Held and Freedman, 1963) have found aftereffects only with sensorimotor integration, which may then lead to motor adaptation. In principal accordance, aftereffects that occur in motor control when the visual or mechanical variables of the targets are perturbed in a systematic manner can be used to test successful motor adaptation (Dutta et al., 2014a). Therefore, controlled variability can be introduced in the form of pseudorandomly interspersed catch trials in the otherwise predictable visuomotor task where the parameter ε_*affected*_ that maps the effect of recorded *CoP_affected_* excursions of the affected side on the cursor acceleration (Equation 5) can be perturbed. Thus, catch trials are proposed to be a reasonable method of exaggerating performance errors during the visuomotor task without disrupting the predictive process. Therefore, the subjects should correct both their own prediction errors and the artificially induced errors resulting from the catch trials in the same manner. It is postulated that in case of successful trial-by-trial adaptation during operant conditioning, the subject should greatly change their CoP excursion ${\left[\begin{array}{c} x\\ y \end{array}\right]}_{CoP_{affected}}$ (Equation 5) on the next trials to catch trial in response to the unusually large error in the catch trial.

### Preliminary evidence: effects of bihemispheric tDCS for the posterior parietal cortex

**Figure 9:**
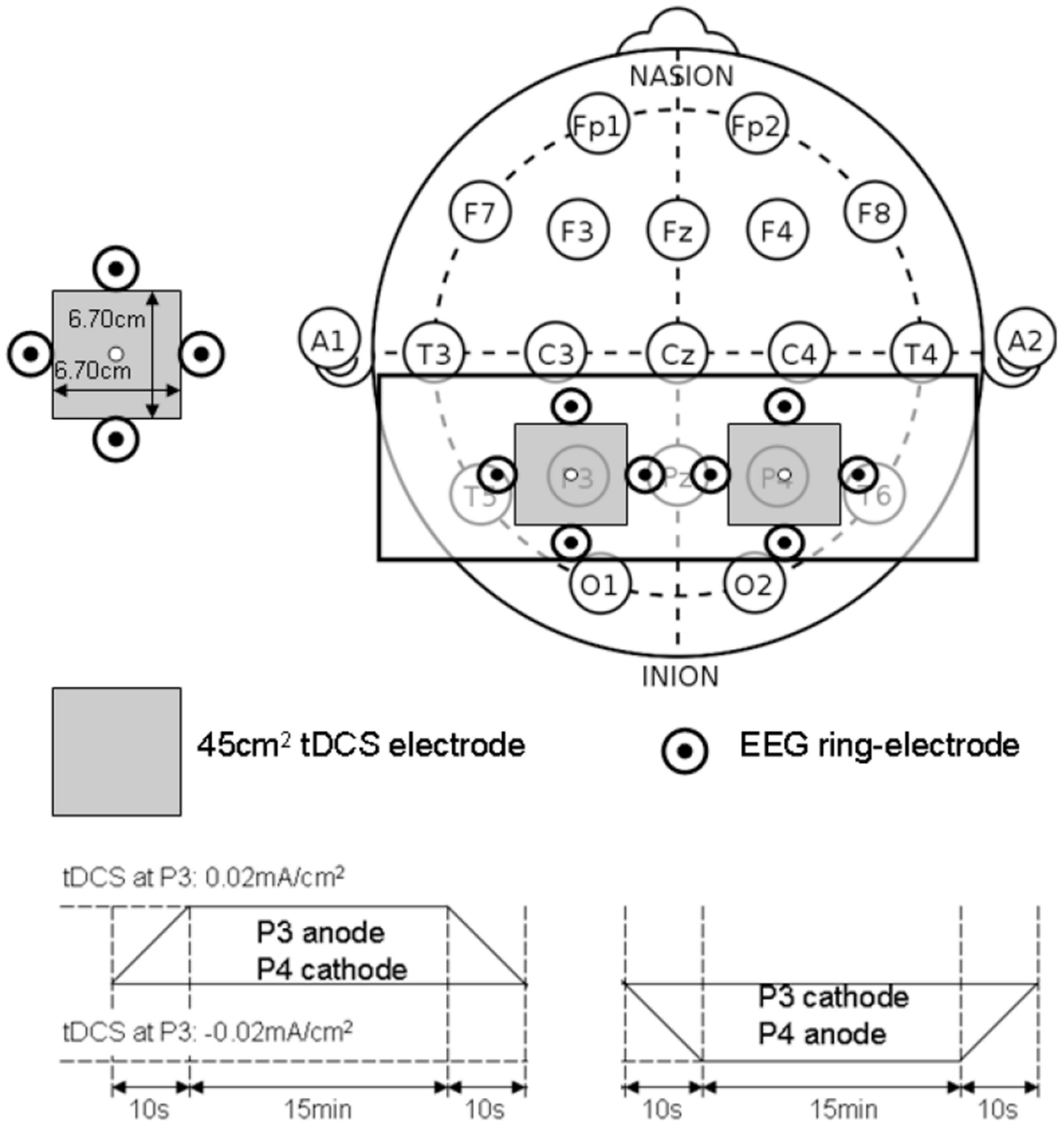
**Bihemispheric tDCS protocol for the posterior parietal cortex**.

The PPC may play a critical role in sensorimotor transformations linking perception to action during quiet standing in terms of CoP trajectory (and stabilogram) (Dutta et al., 2014c). The proof-of-concept pilot study was based on our prior work (Dutta et al., 2014c) where five healthy right-leg dominant male subjects aged between 24 and 46 years were evaluated under two conditions—right-cathodal vs. left-cathodal—tDCS with a pair of 6.7 × 6.7 cm saline-soaked sponge-rubber electrodes (see Figure 9). The current was 1 mA applied for 15 min such that the current density (0.02 mA/cm^2^) was in agreement with Wright and Krekelberg (2013) but lower than our prior work (0.06 mA/cm^2^) (Dutta et al., 2014c). The CoP measurements were made during rest periods of quiet standing for 3 min, just before and immediately after the completion of the tDCS sessions. The study design was repeated-measure, randomized-order with sufficient (1 week) “wash-out” time in between the sessions. Paired *t*-tests (Matlab “ttest” function, The Mathworks, Inc., USA) were performed to compare the impact of right-cathodal vs. left-cathodal for the % post-tDCS change in the centroid of the CoP from baseline (pre-tDCS) values. Indeed, right-cathodal (P4 cathodal, P3 anodal) shifted the CoP centroid toward left by 14 ± 8% and left-cathodal (P4 anodal, P3 cathodal) shifted the CoP centroid toward right by 11 ± 9%. Consequently, a statistically significant (*p* < 0.1) difference was found between right-cathodal vs. left-cathodal tDCS. Since weight bearing on the paretic lower extremity and transfer of weight from one lower extremity to the other are important goals of stroke rehabilitation (De Nunzio et al., 2014), tDCS-facilitated amelioration of post-stroke limb loading asymmetry during biofeedback rehabilitation may improve performance of many functional activities.

## Discussion

The degree to which voluntary guided reaching movements are planned in advance or adapted online is still under investigation. Most well-known models such as the “feedforward models” assume that when motor commands are planned, the outcome of the movement is predicted by the current position of the limbs (Desmurget and Grafton, 2000). According to the “feedforward models” for the visuomotor task, the predicted position of the cursor is compared with the actual position of the cursor with respect to the reaching goal and then online-corrected if the parameters deviate due to noise (e.g., process and observation noise). Thus, a subjects' internal model of the visuomotor task has to be able to adapt to the new dynamics of the environment (Shadmehr and Mussa-Ivaldi, 1994). In fact, it has been proposed that the P300, an ERP component with a parietal scalp distribution, reflects the updating of an internal model of the movement environment that is used to help to plan and execute future motor output (Krigolson et al., 2008). Correspondingly, lesions in the human PPC can lead to complex syndromes consisting of an inability to attend, perceive and react to stimuli in the visual field contralateral to the lesion, an inability to voluntarily control the eye gaze, and an inability to coordinate visually elicited movements (Hyvärinen, 1982; Caminiti et al., 2010; Hwang et al., 2012; Wilke et al., 2012). A recent work demonstrated that in the resting brain, monocephalic anodal tDCS over PPC areas altered ongoing brain activity, specifically in the alpha band rhythm (Spitoni et al., 2013), which may facilitate updating of a deficient internal model during post-stroke rehabilitation. Here, timing of tDCS with respect to the rehabilitation task is critical (Stagg et al., 2011) since regulatory metaplastic mechanisms exist to modulate the effects of a stimulation intervention in a manner dependent on prior cortical excitability, thereby preventing destabilization of existing cortical networks. In our study, the strongest change occurred in the first 2 min after the stimulation ended. Spitoni et al. (2013) found that the tDCS aftereffects diminished systematically and suggested that tDCS affects EEGs immediately after stimulation. Our preliminary study (Dutta and Nitsche, 2013) supported this notion that tDCS affects EEGs immediately after stimulation where Stagg et al. (2011) showed that the application of tDCS during an explicit sequence-learning task led to modulation of behavior in a polarity specific manner: relative to sham stimulation, anodal tDCS was associated with faster learning and cathodal tDCS with slower learning. However, application of tDCS prior to performance of the sequence-learning task led to slower learning after both anodal and cathodal tDCS (Stagg et al., 2011). Based on these prior works that showed that anodal tDCS interacts with subsequent motor learning in a metaplastic manner and suggested that anodal stimulation modulates cortical excitability in a manner similar to motor learning (Stagg et al., 2011), a closed-loop feedback control of bihemispheric tDCS for PPC is proposed during visuomotor task performance, as illustrated in Figure 8.

The goal of this hypothesis and theory paper was to examine prior works for a conceptual review to make a case for multi-level electrotherapy toward post-stroke balance rehabilitation. Under this multi-level electrotherapy concept, both the cortical control of NMES assisted visuomotor task and the motor adaptation toward balance rehabilitation are facilitated with an adjuvant treatment with NIBS. Such a re-conceptualization of electrotherapy approaches, where one (NIBS) is facilitating the other (NMES) toward a common goal (motor learning), could help to push forward electrotherapy for neurorehabilitation.

### Conflict of interest statement

The authors declare that the research was conducted in the absence of any commercial or financial relationships that could be construed as a potential conflict of interest.

## Footnotes

^1^http://www.mathworks.com/matlabcentral/fileexchange/file_infos/32493-eogui-a-software-to-analyze-electro-oculogram-eog-recordings. Accessed April 2.
